# Advances and Challenges in *Aeromonas hydrophila* Vaccine Development: Immunological Insights and Future Perspectives

**DOI:** 10.3390/vaccines13020202

**Published:** 2025-02-18

**Authors:** Kavi R. Miryala, Banikalyan Swain

**Affiliations:** Department of Infectious Diseases & Immunology, College of Veterinary Medicine, University of Florida, Gainesville, FL 32608, USA

**Keywords:** aquaculture, *Aeromonas hydrophila*, infectious diseases in fish, fish vaccination, fish pathogens, fish immunology, bacterial diseases in fish, fish vaccines

## Abstract

*Aeromonas hydrophila* presents a significant threat to global aquaculture due to its ability to infect freshwater and marine fish species, leading to substantial economic losses. Effective mitigation methods are essential to address these challenges. Vaccination has emerged as a promising strategy to reduce *A. hydrophila* infections; however, it faces several obstacles, including variability in immune responses, pathogen diversity, and environmental factors affecting vaccine efficacy. To enhance vaccine performance, researchers focus on adjuvants to boost immune responses and develop multivalent vaccines targeting multiple *A. hydrophila* strains. Tailoring vaccines to specific environmental conditions and optimizing vaccination schedules can further address the challenges posed by pathogen diversity and variable immune responses. This review provides an in-depth analysis of the immunological hurdles associated with *A. hydrophila* vaccine development. Current vaccine types—live attenuated, inactivated, subunit, recombinant, and DNA—exhibit diverse mechanisms for stimulating innate and adaptive immunity, with varying levels of success. Key focus areas include the potential of advanced adjuvants and nanoparticle delivery systems to overcome existing barriers. The review also highlights the importance of understanding host–pathogen interactions in guiding the development of more targeted and effective immune responses in fish. Complementary approaches, such as immunostimulants, probiotics, and plant-based extracts, are explored as adjuncts to vaccination in aquaculture health management. Despite notable progress, challenges remain in translating laboratory innovations into scalable, cost-effective solutions for aquaculture. Future directions emphasize the integration of advanced genomic and proteomic tools to identify novel antigen candidates and the need for industry-wide collaborations to standardize vaccine production and delivery. Addressing these challenges can unlock the potential of innovative vaccine technologies to safeguard fish health and promote sustainable aquaculture practices globally.

## 1. Introduction

*A. hydrophila* is a Gram-negative, chemo-organoheterotrophic, facultatively anaerobic, rod-shaped bacterium commonly found in freshwater environments, where it acts as a lethal pathogen, particularly in domestic and wild fish species [1,2,3]. Beyond fish, its host range extends to humans, animals, birds, and marine and freshwater reptiles [4,5,6]. This review focuses on its implications for aquaculture, where it poses significant threats to fish populations. *A. hydrophila* causes diseases such as peritoneal inflammation, bacteremia, meningitis, cholera-like enteric disease, hemolytic uremic syndrome, necrotizing fasciitis, gastroenteritis, motile Aeromonas septicemia (MAS), and ulcerative infections [7,8,9,10,11]. Its virulence arises from factors such as lipopolysaccharides, outer membrane proteins (OMPs), pili, flagella, the type III secretion system (T3SS), and extracellular toxins like aerolysin of *aerA*, hemolysins of *hlyA*, and cytolytic enterotoxins of *act*, which facilitate host invasion and immune evasion. In aquaculture, these factors contribute to high morbidity and mortality rates, severely compromising sustainability [3,12,13,14,15,16]. These virulence factors enable *A. hydrophila* to invade host tissues and evade immune defenses, resulting in various health complications in infected species [16,17]. The bacterium poses significant threats to aquaculture, causing devastating losses due to high morbidity and mortality rates among fish populations [11]. Its infections compromise not only the health of individual animals but also the overall efficiency and sustainability of aquaculture systems. Transmission occurs through direct contact with infected organisms or contamination of water sources, allowing the pathogen to spread within aquaculture environments [18]. However, disease onset is not guaranteed, as immunocompromised individuals are most susceptible to infection. In aquaculture, stressful conditions such as overcrowding, reduced oxygen levels, high organic matter, physical injuries, temperature fluctuations, and industrial pollution increase the prevalence of immunosuppressed individuals, creating ideal conditions for outbreaks. Addressing these stressors is crucial to mitigating the impact of *A. hydrophila* in aquaculture [19,20].

Aquaculture has become a pivotal component of the global economy, significantly enhancing food security worldwide [21,22]. Over the last fifty years, aquaculture fish production has risen steadily, driven by the growing demand for fish as a cost-effective and highly efficient source of animal protein [21,23,24]. A remarkable milestone was achieved in 2022, when aquaculture’s contribution to global aquatic animal production surpassed that of capture fisheries for the first time, highlighting its growing dominance [22]. Additionally, freshwater aquaculture alone accounted for approximately 83% of aquatic animal production from inland waters, further emphasizing its critical role in meeting global protein demands [22]. This shift underscores aquaculture’s capacity to address the rising demand for aquatic foods while alleviating pressure on capture fisheries. However, sustaining this trajectory requires prioritizing sustainable development practices and ensuring equitable benefits for vulnerable communities dependent on these resources [25]. By aligning expansion with these goals, aquaculture can continue to bolster global food systems and foster economic resilience.

Beyond its significant impact on aquatic environments, *A. hydrophila* has been identified as a pathogen in humans, with its presence confirmed in stool samples [3]. The primary transmission route is fecal–oral, often through the consumption of contaminated water or food products such as meat, dairy, shrimp, or fish, many of which originate from aquaculture systems [11,26]. In humans, the bacterium can cause both intestinal diseases, such as gastroenteritis and traveler’s diarrhea, and extra-intestinal conditions, including septic arthritis, skin and wound infections, meningitis, and rapidly progressing septicemia, particularly in immunocompromised individuals [16,27]. These human health risks underscore the urgency of containing *A. hydrophila* in aquaculture systems. This containment can be achieved through improved care practices, strict biosecurity measures, and the development and application of effective vaccines to mitigate both environmental and zoonotic transmission risks.

A significant aspect of problems is in the treatment methodologies. In the past, farmers used traditional antibiotics, including amikacin, ampicillin, cefotaxime, amoxicillin, trimethoprim-sulfamethoxazole, erythromycin, and streptomycin, along with others [28,29]. However, the improper use and excessive reliance on these antibiotics have resulted in a rise in antibiotic-resistant cases among *A. hydrophila* in aquaculture environments [30]. In response, farmers increased the doses of these antibiotics, making them almost ineffective from specific strains over time [30,31]. Studies suggest that some antibiotics are still viable in low doses, including thiamphenicol and florfenicol; however, these variations will soon face the same challenges of antibiotic resistance [32]. In response, multi-drug combinations have been presented for treatment, but *A. hydrophila* has been shown to contribute almost 90% of multi-drug resistance in tested isolates, making antibiotics not viable for future innovations in treatment [33].

The nature of the bacterium makes *A. hydrophila* an opportunistic pathogen that is seeing an increasing resistance to antibiotics, making the development of effective vaccines ever more crucial for sustainable aquaculture. Currently, vaccine development focuses on live attenuated, inactivated, subunit, and recombinant vaccines, with advancements in modern biotechnological vaccines accelerating efficiency, which aids in filling the gap for increased global demands for fish consumption [34]. These four vaccine categories show varying efficacy due to their mechanisms, and there is an increasing pursuit of multivalent vaccines, ideal for aquaculture as they protect against multiple pathogens in a single dose [35]. Methods of vaccine delivery, immune responses, effectiveness, adjuvant or carrier systems, nanoparticles, and genomic and proteomic approaches will also be expanded on in this review [34,36,37]. Vaccine development has already begun for *A. hydrophila*, with several reputable multivalent vaccines available, despite currently struggling to keep up with strain variability, high development costs, and the complexity of delivering vaccines in aquaculture environments [38]. Continued research, funding, and collaboration between the scientific community and the aquaculture industry will aid in advancing effective vaccine solutions to meet global demands while encouraging sustainability. This review delves into the immunological mechanisms underlying current vaccines, the challenges faced, recent innovations, and future directions in vaccine development against *A. hydrophila.*

## 2. Immunological Basis of Vaccination

The fish immune system consists of both innate and adaptive components that work synergistically to provide effective protection against pathogens [39]. Vaccination is a critical strategy for stimulating the immune system to recognize and combat *A. hydrophila* during subsequent exposures, offering a promising alternative to combat antibiotic-resistant bacterial strains [40]. It initiates a complex interplay between the innate and adaptive immune systems, effectively “training” the adaptive immune response to recognize specific pathogens while also activating and modulating components of innate immunity [41]. The inclusion of specific adjuvants in vaccines further enhances their efficacy by focusing on promoting either innate or adaptive immune responses, depending on the immunological target [42,43]. The immunological basis of vaccination lies in its ability to stimulate a dual response: the innate immune system provides rapid, nonspecific defense, while the adaptive immune system generates long-lasting, pathogen-specific immunity. This mechanism is particularly critical for *A. hydrophila*, as vaccine subtypes and adjuvants are designed to overcome challenges like strain variability, ensuring broad-spectrum protection and enhanced vaccine efficacy [44].

### 2.1. Innate Immunity

**Figure 1:**
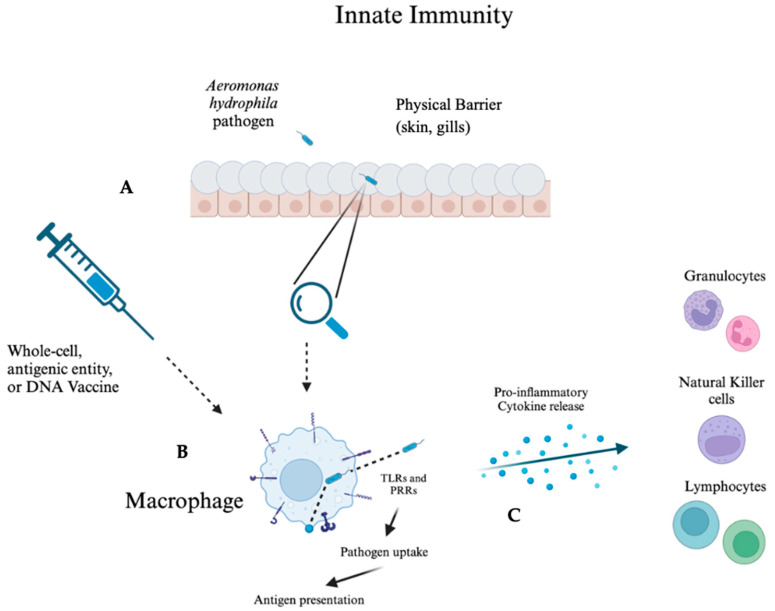
**An overview of the innate immune response to *Aeromonas hydrophila* in fish.** (A) Pathogen entry and vaccination: *A. hydrophila* encounters the physical barriers of the skin and gills, which are covered with mucus, serving as the first line of defense. The circles shown represent the scales, while the cells below represent the epidermis of the fish, which contain mucus-secreting goblet cells that consist of macrophages for pathogen uptake (B) [54]. Breach of these barriers allows the pathogen to enter. The alternative introduction of pathogenic entities through a vaccine—including whole-cell, antigenic entities, or DNA vaccines—primes the immune system to recognize and combat the pathogen. (B) Macrophage activation and antigen presentation: Upon pathogen entry, macrophages recognize the pathogen through TLRs and PRRs. This interaction triggers pathogen uptake and antigen presentation, initiating the immune response cascade. These antigens are then functional in adaptive immunity (Figure 2). (C) Cytokine release and recruitment of immune cells: Activated macrophages release pro-inflammatory cytokines, which recruit granulocytes, natural killer cells, and lymphocytes to the site of infection. These immune cells work in coordination to eliminate the pathogen and restore homeostasis. The mechanisms of this figure are based on literary data, which was modeled on BioRender.com (accessed on 12 November 2024).

Innate immunity involves non-specific immune responses and includes physical barriers in fish, such as the skin and gills. Cellular responses also play a crucial role, where pathogen-associated molecular patterns (PAMPs) are recognized by pattern recognition receptors (PRRs) and Toll-like receptors (TLRs), triggering phagocytosis by macrophages and neutrophils, as well as the release of cytokines to initiate inflammation (Figure 1) [39,40,45,46,47]. Lysozymes and antimicrobial peptides work synergistically to trap and neutralize pathogens within the physical barrier [41,47]. These mucosal surfaces are dynamic and adapt to environmental factors, modifying the secretion of immune molecules and the composition of the microbiota [47,48]. This adaptability influences the immune response and significantly affects the fish’s susceptibility to *A. hydrophila*. Vaccinated fish, for example, showed an increase in lysozyme concentration in skin mucus, which acts as a physical barrier by breaking down bacterial cell walls [49]. Additionally, heat-inactivated and formalin-inactivated *A. hydrophila* vaccines have demonstrated the ability to enhance superoxide anion production, complement activity, phagocytic function, and lysozyme activity, indicating that these vaccines effectively stimulate PRRs and TLRs [50].

Vaccination, coupled with the introduction of adjuvants, significantly enhances cellular responses in innate immunity. Genetic variability, delivery methods, vaccine variants, and the virulence of the pathogen strain can influence the outcomes of innate immune mechanisms, which are generally positive in successful vaccines [43]. Upon administration of a vaccine against *A. hydrophila*, components of the innate immune system, such as dendritic cells and macrophages, become activated by PRRs that recognize PAMPs present in the inactivated bacterial antigens or whole-cell variants [46,51]. These innate immune cells process and present *A. hydrophila* antigens to T cells, bridging the gap in the adaptive immune system [46,47]. Cytokines and chemokines released by these innate immune cells shape the adaptive response by influencing T cell differentiation and B cell activation, critical for producing specific antibodies against *A. hydrophila* [52,53]. Through this coordinated effort, the innate system not only provides immediate, non-specific defense via mechanisms such as enhanced lysozyme activity but also establishes a foundation for the adaptive immune system to develop a more targeted and long-lasting response [53]. This interplay ensures the fish is primed with memory B and T cells, enabling a robust response upon future pathogen exposure.

### 2.2. Adaptive Immunity

Adaptive immunity in fish provides a highly specific defense against *A. hydrophila*, resulting in the production of pathogen-specific antibodies and the development of immunological memory. However, the mechanisms by which *A. hydrophila* modulates the immune response in fish remain incompletely understood. During primary infection, *A. hydrophila* often suppresses immune functions, as evidenced by reduced phagocytic activity, diminished respiratory burst, and decreased leucocyte peroxidase content in several fish species [55,56,57] Studies also suggest that the bacterium can evade host defenses by producing extracellular products such as proteases and toxins, which further impair immune signaling pathways and inhibit macrophage function [58,59]. Despite these challenges, adaptive immunity plays a crucial role in overcoming infection, with vaccine-mediated immunity helping to restore immune function and enhance specific responses against *A. hydrophila* antigens [39].

**Figure 2:**
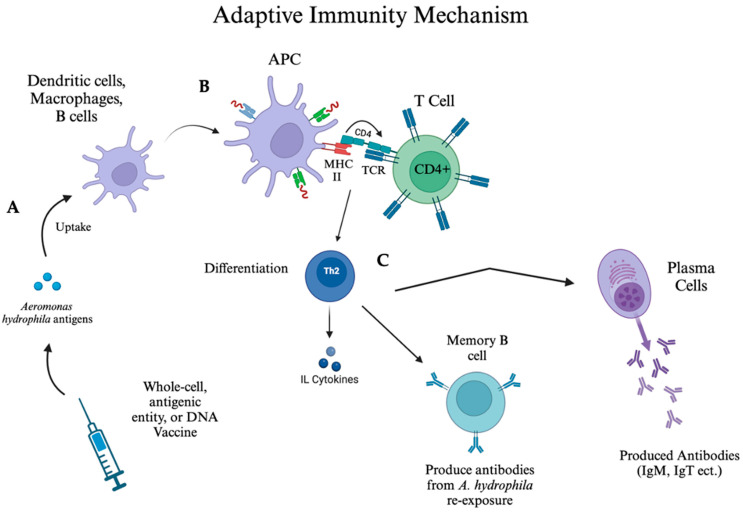
**Overview of adaptive immunity mechanism against pathogens such as *Aeromonas hydrophila*.** (A) Antigen introduction through vaccination or innate immunity: Vaccines—whole-cell, antigenic entity, or DNA-based—introduce antigens to immune system. Alternatively, antigens from innate immune response (Figure 1) can stimulate adaptive immunity. (B) Antigen-presenting cell (APC) formation and T cell interaction: Antigens are taken up by dendritic cells, macrophages, or B cells, which then process and present antigens via major histocompatibility complex II (MHC II) to CD4^+^ T cells through T cell receptor (TCR) and cluster of differentiation 4 (CD4) co-receptor [37,66,67]. This interaction activates T cells, initiating adaptive immune response. (C) Th2 cell differentiation and antibody production: Activated T cells differentiate into Th2 cells, which release cytokines such as interleukins (IL) [61,62,66]. These cytokines stimulate the formation of memory B cells and plasma cells. Memory B cells ensure rapid antibody production upon re-exposure to pathogens such as *A. hydrophila*, while plasma cells produce antibodies, including IgM and IgT, to neutralize pathogens [61,62,66]. This image was modeled on BioRender.com (accessed on 12 November 2024) and is based on literary data.

Following vaccination or natural infection with *A. hydrophila* and similar pathogens, memory B and T cells are formed, providing long-term immunity [60,61]. Key players in the adaptive immune response include Immunoglobulin M (IgM), the primary antibody that targets the pathogen [39,40,62]. However, IgM provides early but short-term immunity initiated by vaccination, preventing long-term vaccine response, which is maintained by Immunoglobulin G (IgG), an antibody that is absent in fish [63]. T and B lymphocytes are also crucial, as in cell-mediated response, T-helper cells (CD4^+^) assist in activating B cells to produce antibodies and memory B cells (Figure 2) [60,64,65]. Tumor necrosis factors (TNFs) are involved in the regulation of the functionality of these B cells [60,64,65]. Additionally, cytotoxic T cells (CD8^+^) are responsible for targeting and eliminating infected cells [37,61,66]. The presence of these cells and mechanisms in the fish immune response is paralleled to that of human adaptive immunity, which is biochemically similar in fish species.

In fish, the cytotoxic CD8^+^ T cell mechanism relies on MHC I molecules presenting intracellular antigens to CD8^+^ T cells, enabling the recognition and elimination of infected or abnormal cells [37,66,67]. This process is further supported by differentiated type I T-helper cells (Th1), which release cytokines such as IL-2 to stimulate CD8^+^ T cell proliferation [68]. IFN-γ and TNF-α, secreted by TH1 cells, play pivotal roles in enhancing MHC I expression on infected or abnormal cells, amplifying the inflammatory response [69,70]. This upregulation of MHC I facilitates the recognition of intracellular antigens by CD8^+^ T cells, ensuring efficient targeting and destruction of pathogen-infected or dysregulated host cells [37,69]. The coordinated interactions between TH1 cytokines and CD8^+^ T cells represent synergy within the immune system, promoting an effective cytotoxic response to eliminate *A. hydrophila* while maintaining immune regulation [37,66,67]. Specific antibody responses, such as that of pathogen-specific IgM, help neutralize the bacteria, while cytokines produced by lymphocytes influence the differentiation of T cells into type I T-helper cell (Th1) and type II T-helper cell (Th2) pathways (Figure 2) [61]. Studies also highlight the importance of memory B cells in the humoral response, which helps to generate a faster response upon re-exposure to a pathogen. As a result, the secondary immune response is typically quicker and stronger than the initial response due to the “primed” nature of the memory cells [61,62,66].

However, most vaccine-based studies on *A. hydrophila* have shown limitations in assessing adaptive cell-mediated immunity mechanisms. In contrast, measuring humoral and innate immune responses has been consistently emphasized and extensively explored [34,71]. Despite these limitations, some studies have demonstrated that vaccination can induce significant antigen-specific lymphocyte proliferation and other antigen-specific responses, particularly when using somatic and OMP antigens. This antigen specificity, driven by cell-mediated responses, is a hallmark of adaptive immunity, highlighting the critical roles of T cells and lymphocyte proliferation in orchestrating a targeted immune defense [72,73,74]. More specifically, antibodies such as IgM were significantly elevated in most vaccinated studies, which will be shown throughout this review. Adaptive immunity was also marked by elevated levels of specific antibodies such as Immunoglobulin T (IgT) in both blood serum and mucosal surfaces, specifically through memory B cells [49,75].

## 3. Current Vaccines for *Aeromonas hydrophila*

Vaccines developed for *A. hydrophila* include live attenuated, inactivated, subunit, recombinant, and plasmid DNA variations, each capable of inducing both humoral and cell-mediated immunity. These vaccines differ in the specific components of *A. hydrophila* they present, with mechanisms relying on targeted antigen presentation or the inactivation of whole-cell components to reduce virulence while enhancing antibody production [76,77,78]. The efficacy of these vaccines in stimulating innate and adaptive immune responses is typically measured through relative percent survival (RPS) in fish challenged with *A. hydrophila* strains [79]. Various studies have explored vaccine mechanisms and cellular components specific to different *A. hydrophila* strains and their effects on diverse aquaculture species [37]. Efforts to optimize survival rates have focused on factors such as antigen concentrations, delivery methods, adjuvants, and post-challenge periods. The highest RPS outcomes observed in the reviewed studies will be highlighted in the following sections.

### 3.1. Live Attenuated Vaccines

Live attenuated vaccines use an auxotrophic mutant that increases antibody production, activates immune-related genes, and enhances non-specific defenses like lysozyme and antiprotease activities, which leads to reduced bacterial virulence [80,81]. The development of these vaccines for *A. hydrophila* has focused on genetic modifications targeting specific virulence- and growth-associated genes, resulting in the attenuation of a mutant strain that projects reduced pathogenicity while retaining immunogenic properties. Gene deletions in studies include a five-gene deletion strain targeting *aerA*, *hly*, *ahp*, *alt*, and *ast* in *Ctenopharyngodon idella* (grass carp), the deletion of the *crp* and *fur* genes in *Carassius carassius* (crucian carp), and the *aroA* gene in *Oncorhynchus mykiss* (rainbow trout) [25,82,83]. The multi-gene deletion strain achieved a 240-fold reduction in LD50 compared to wild-type strains and survival rates of over 70% post-challenge [84]. The *fur* gene encodes the Fe^2+^ regulatory protein, while the *crp* gene codes for the cAMP receptor protein [82]. Deletion of these genes was achieved through a suicide plasmid method that resulted in the upregulation of immunogenic enzymes of superoxide dismutase, lysozyme, alkaline phosphatase, and acid phosphatase while eliciting high levels of IgM antibodies upon vaccination [82]. Here, RPS was recorded to be 83.3% for the Δ*fur* strain and 73.3% for the Δ*crp* strain [82]. The *aroA* gene was inactivated using homologous recombination and was also facilitated by a suicide vector [83]. The result of this vaccine was a 75% RPS from the wild-type challenge resulting from an increase in antibody titers and non-specific immune markers, such as IgM [83].

Attenuation through rifampicin-induced mutation of the XX1LA strain in *Cyprinus carpio* (common carp) demonstrated effective immune activation, while transposon insertion mutagenesis of the *A. hydrophila* J-1 strain to eliminate protease, hemolysin, amylase, and DNase activity was tested in *Xiphophorus helleri* (swordtail fish) [52,85]. These modifications represent diverse approaches to designing live attenuated vaccines specific to the pathogen and the host species. The rifampicin-mutated inactivated vaccine revealed an upregulation of pro-inflammatory cytokines (IL-1β) and anti-inflammatory cytokines (IL-10) in the spleen and liver, IgM anybody titer, and lysozyme serum activity, with an RPS of 83.7% [52]. Meanwhile, the J-1 strain provided a protection rate of 64%, which correlated with increased phagocytic activity [85].

These findings consistently demonstrated that live attenuated vaccines elicit robust humoral and cellular immune responses, achieve high survival rates, and offer strain-specific protection, making them highly effective tools for controlling *A. hydrophila* in aquaculture. They do require a more sophisticated approach compared to vaccines presenting strictly antigenic components; however, their whole-cell approach allows for long-term immunity, minimizing the need for boosters.

Advancements in recombinant attenuated vaccine systems, such as those developed for *Edwardsiella piscicida*, offer promising insights for addressing challenges posed by *A. hydrophila*. The RAEV system integrates cutting-edge technologies to achieve regulated delayed attenuation [86] and environmental containment, ensuring safety and efficacy. One example involves the deletion of *asdA* in *E. piscicida*, requiring diaminopimelic acid (DAP) supplementation or a plasmid carrying the *asdA* gene for bacterial survival. This balanced lethal system enables the synthesis and delivery of the protective *Ich* antigen IAG52B [87]. A regulated murA system also introduces controlled cell wall lysis by replacing the native promoter with an arabinose-regulated *araC* P_araBAD_ cassette. This ensures bacterial survival only in the presence of arabinose and induces lysis post-colonization, preventing environmental persistence. These systems stimulate robust immune responses, including elevated cytokine expression (TNF-α, IL-1β, IL-6, IL-8) and systemic and mucosal IgM production, offering targeted protection against bacterial and parasitic pathogens [87,88].

The application of these technologies highlights their potential as versatile platforms for designing vaccines against multiple pathogens, including *A. hydrophila*. Their ability to induce strong immune responses, prevent environmental contamination, and remain sensitive to antibiotics aligns with the goals of sustainable aquaculture practices. Adapting such advanced vaccine strategies could revolutionize the control of *A. hydrophila* and other aquaculture pathogens, mitigating economic losses while promoting environmental responsibility.

### 3.2. Inactivated Vaccines

Inactivated vaccines against *A. hydrophila* utilize heat or chemically killed bacterial cells to stimulate humoral and innate immune responses. These vaccines effectively increase antibody production, activate immune-related genes, and enhance non-specific defense mechanisms, such as lysozyme activity, to protect against *A. hydrophila* infections [49,52,79,89,90]. As the bacteria are inactivated, they cannot replicate, which makes these vaccines inherently safe. However, they primarily stimulate humoral immunity and often require the use of adjuvants to enhance the immune response. The majority of current *A. hydrophila* studies employ the use of diluted formalin and even formaldehyde to inactivate a virulent strain of the bacterium, maintaining the whole-cell features.

Intraperitoneal vaccination with a formalin-killed *A. hydrophila* suspension in *Piaractus mesopotamicus* (pacu) resulted in significantly elevated antibody titers in both blood serum and skin mucus, alongside increased lysozyme activity in mucus [49]. These effects persisted up to 84 days post-vaccination, and booster doses administered via immersion further enhanced immune responses [49]. The RPS was recorded at 31.33% in intraperitoneally vaccinated fish after 84 days, highlighting the potential of combined vaccination strategies to improve both mucosal and systemic immunity [49]. A study using immersion vaccination with an inactivated bacterin through the addition of buffered formalin in *Ictalurus punctatus × Ictalurus furcatus* (hybrid catfish) offered sustained protection for at least seven weeks post-vaccination, mitigating acute mortality caused by epidemic vAh isolates in the U.S. catfish industry [89].

In *Oreochromis niloticus* (tilapia), formalin-killed vaccines with Freund’s complete adjuvant achieved a 100% relative level of protection within two weeks post-vaccination [90]. In *Megalobrama amblycephala* (Wuchang bream), vaccines inactivated with 0.3% formaldehyde significantly enhanced the expression of immune-related genes, such as IL-1β, IL-6, IL-10, TNF-α, MHC I, and MHC II, as well as the activity of antimicrobial enzymes in the liver and serum [79]. These changes contributed to improved bactericidal abilities and increased survival rates during experimental infection challenges, making these vaccines a promising option for controlling *A. hydrophila* outbreaks in this species [79]. Finally, a formalin-killed vaccine in *Cyprinus carpio* (common carp) moderately increased immunity, although RPS was limited to 37.2% compared to 83.7% for the counterpart live attenuated vaccine [52]. While the efficacy of inactivated vaccines in this case was lower, they still provided measurable immune protection, demonstrating their value as a safer alternative to live vaccines in certain aquaculture settings [52].

These studies collectively illustrate that inactivated vaccines are effective tools for preventing *A. hydrophila* infections across a range of fish species. They offer practical advantages such as ease of production and reduced regulatory hurdles compared to live attenuated vaccines, although their efficacy can vary depending on the host species, vaccine formulation, and delivery method. Optimization tailored to specific aquaculture environments and pathogens remains critical to maximizing the effectiveness of these vaccines. In some cases, booster doses are required when the primary immune response begins to decline, as shown by [49,89].

### 3.3. Subunit Vaccines

The mechanism of a subunit vaccine’s function can vary but generally involves isolating and purifying specific components of a pathogen, such as OMPs to stimulate humoral and cellular immunity, inducing specific antibodies, and enhancing immune responses [79]. Antigens in subunit vaccines are processed by APCs, which are presented to T-helper cells that further stimulate B-cells to produce antibodies (Figure 2) [91]. This reduces bacterial load and activates immune-related genes. A key advantage of this mechanism is that it eliminates the risk of infection since pathogenic components are not present and contamination by other microorganisms is impossible, making them a safer option [34]. Research focused on the protein products of *A. hydrophila is* essential to the production of other variants of native subunit vaccines [34].

Some specific antigenic entities that have been used include OMPs and lipopolysaccharides (LPS) in *Ctenopharyngodon idella* (Grass Carp), OMPs in *Carassius auratus* (goldfish), Glycoprotein-based native subunit in *Oncorhynchus mykiss* (rainbow trout), Maltoporin (46 kD) in *Anguilla anguilla* (European eel), OmpA in *Megalobrama amblycephala* (Wuchang bream), extracellular products (ECPs) in *Ictalurus punctatus* (channel catfish), and *Carassius carassius* (crucian carp) [34,79,92,93,94,95,96,97].

There have been several other variations of subunit vaccines in different fish species; however, the use of these proteins and products of *A. hydrophila* is generally the most common. Within these studies, the RPS was generally above 50%, demonstrating the effectiveness of the vaccine [34,79,92,93,94,95,96,97]. Given this, identifying the most immunogenic antigens across all *A. hydrophila* vaccines is difficult due to the variance across species and strains of the bacterium. The OMPs of *A. hydrophila* are a widely applied antigen, as they are highly conserved across various strains, making them excellent targets for developing subunit vaccines that offer broad-spectrum immune protection [79,98,99]. Despite this, the subunit vaccines sometimes have lower immunogenicity compared to live vaccines, with this limitation arising because subunit vaccines typically display fewer antigens compared to whole-cell vaccines, resulting in reduced replication or variety in antigen exposure [100]. In the specific case of the OmpA subunit vaccine, it has been shown to have better immune protection than the inactivated vaccine when the RPS was evaluated and compared, despite both vaccines displaying excellent immune protection in *M. amblycephala* [79].

Generally, to maintain long-lasting protective immunity, several booster doses, as well as several adjuvants, are required [100]. Also, the necessity of cold chain storage and precise formulation can limit accessibility and economic feasibility in fish aquaculture, making subunit vaccines primarily considered for high-value fish species or in situations where specific disease pressures justify the added expense [100].

### 3.4. Recombinant Vaccines

The last subtype is the recombinant vaccine which utilizes specific proteins, such as the S-layer protein and OMPs of different *β*-barrels that are produced through recombinant DNA technology [77]. These are much like the subunit vaccines, by offering specific antigens to stimulate immune response; however, the key difference lies in the methods of acquiring these proteins. Recombinant vaccine development involves the process of polymerase chain reaction (PCR) and gel electrophoresis to amplify and analyze the DNA [71]. Following this, a protein-specific gene from *A. hydrophila* is cut with restriction enzymes and ligated into a plasmid or other vector compatible with the host organism used for protein expression [99]. Transformation then occurs, incorporating the gene into another organism to elicit the production of these antigens for vaccine formulation. In the case of *A. hydrophila* vaccines, various strains of *Escherichia coli* and even *Lactococcus lactis* are often used as the genetically modified organism, which are cultured and usually treated with operon inducers to promote protein expression [34,71,77,99,101,102,103,104,105,106,107,108,109,110,111,112,113]. These recombinant proteins produced are then extracted and purified using protein-specific His-tags or other methods [71]. The accumulation of these methods results in the process of cloning the gene, encoding the desired protein into an expression vector, and then expressing and purifying the protein in a host organism that can produce these specific antigens [71,77].

The current recombinant antigens produced or genes cloned across *A. hydrophila* vaccine studies, through variations of the procedures mentioned, include aerolysin D1 and D4 genes cloned in tilapia, rOmpR in *Labeo rohita* (Rohu), iron-regulated OMPs in *Danio rerio* (zebrafish), and *maltoporin* (46 kD) in *Anguilla anguilla* (European eel) [34,71,77,99,101,102,103,104,105,106,107,108,109,110,111,112,113]. Additional recombinant antigens include the *aerA* recombinant protein in *Ctenopharyngodon iella* (grass carp), fimbrial proteins (*FimA*, *Fim*, *FimMrfG*, and *FimOM*) in *Ictalurus punctatus* (Channel Catfish), *OmpW* in *Labeo rohita* (Rohu), and S-layer proteins in *Cyprinus carpio* (common carp). Furthermore, recombinant hemolysin co-regulated protein (*Hcp*) is utilized in *Cyprinus carpio* (common carp), with rOmp48 tested in *Labeo rohita* (Rohu), *Omp38* in *Megalobrama amblycephala* (Wuchang bream), and OMPs (*Aha1* and *OmpW*) in *Cyprinus carpio* (common carp) [34,71,77,99,101,102,103,104,105,106,107,108,109,110,111,112,113].

The immunological consequences of these vaccines were specifically shown with the SWCNTs-aerA recombinant vaccine, which showed significant upregulation of the IgM gene that produces the primary antibody, resulting in the activation of innate immunity [105,114,115,116]. Further genes found to elicit immunity included IFN-I, TNF-α, and IL-8, which were also upregulated, suggesting that innate immunity activation supports the initiation of adaptive immune responses [105,114,115,116]. The recombinant *Hcp* in *Cyprinus carpio* (common carp) found the vaccine also elevated IgM antibody levels, which directly correlated with increased survival rates [112]. Fish vaccinated with *Hcp* demonstrated a survival rate of 46.67% over a 10-day challenge, in contrast to a 7.14% survival rate in unvaccinated fish. Cytokine levels, including IL-1β and TNF-α, were found to be significantly upregulated in vaccinated common carp, enhancing immune resilience in kidney, spleen, and gill tissues [112]. Iron-regulated OMPs in *Danio rerio* (zebrafish) demonstrated increased immunogenicity when expressed under iron-limited conditions, as these proteins are involved in essential iron transport processes required by *A. hydrophila* for survival [111]. Proteomic analysis revealed that these OMPs effectively stimulate antibody responses and enhance iron homeostasis pathways, correlating directly with increased survival in vaccinated zebrafish following exposure to a virulent *A. hydrophila* strain [111]. Furthermore, recombinant antigens can foster prolonged adaptive immunity through extended antigen exposure. The elevated specific immune responses in *Megalobrama amblycephala* (Wuchang bream), immunized with recombinant Omp38 protein, revealed significant serum IgM antibody levels, increased superoxide dismutase, and lysozyme activities in head kidney lymphocytes [109]. Immunized fish demonstrated a marked increase in phagocytic activity, resulting in superior protective effects compared to inactivated vaccines. This recombinant variant effectively stimulated both specific and non-specific immune responses, enhancing survival rates in vaccinated fish [109]. These findings were often strain-specific and also often applied to the other recombinant vaccine studies previously mentioned [34,71,77,99,101,102,103,104,105,106,107,108,109,110,111,112,113].

The production of recombinant vaccines, including OMPs like Aha1 and OmpW in *Cyprinus carpio* (common carp), was also found to be technically complex and costly [99]. This is due to the high precision required during the expression and purification processes and is consistent across all the mentioned studies [71,77,99,101,102,103,104,105,106,107,108,109,110,111,112,113]. Despite these challenges, such recombinant vaccines provide significant immunoprotective advantages for aquaculture, though their high initial production costs could be a barrier to widespread adoption in some fish farming contexts.

### 3.5. DNA Vaccines

The mechanism of DNA vaccines involves the process of introducing plasmid DNA into cells that contain genes to encode specific antigens or bacterial surface structures from *A. hydrophila* by cloning these components from *A. hydrophila* genome DNA. These vaccines also incorporate specific genes that encode molecules involved in directly stimulating immune response [117]. Once these components are transcribed and translated within the host cell, they directly elicit humoral and cellular immunity, which reduces pathogenicity upon further exposure, as other vaccine variants do [118]. The process of DNA vaccine formulation for *A. hydrophila* requires high-virulence isolates. To obtain these isolates, researchers initially use bioinformatical tools to identify and design primers for the isolates’ genes. The gene is then amplified through PCR, and these antigenic or surface structure components of *A. hydrophila* are obtained, screened, and selected based on their mortality impact and commonality across other strains. The gene can then be inserted into a specific plasmid vector and transformed into *E. coli* or other competent cells. This bacterium is then cultured and screened for successful cloning. The recombinant plasmid produced can be inserted into the targeted host cells, where they can be checked for protein expression using Western blotting or ELISA techniques to confirm the presence of the desired protein. Variations of these methods are also often employed.

Plasmid DNA encodes the gene for the production of recombinant antigens apolipoprotein A1 (pcDNA-ApoA1) in *Ictalurus punctatus* (channel catfish), G-protein coupled receptor 18 (GPR18) (pcDNA-GPR18) in *Ictalurus punctatus* (channel catfish), and yeast-based ovalbumin ompG (pET32a-OVA-ompG) and omp48 (pET32a-OVA-omp48) in *Carassius carassius* (crucian carp) [119,120,121]. The pcDNA-ApoA1 vaccine combined with an adjuvant provided complete (100%) protection to channel catfish against a highly virulent *A. hydrophila* strain while serum lysosome and macrophage activity increased, demonstrated by significantly elevated levels of reactive oxygen species (ROS) and nitric oxide (NO). Of the yeast-based vaccines, the OVA-ompG group elicited stronger specific immune responses compared to the OVA-omp48 group with an RPS of 46% after 21 days [120]. The GPR18 vaccine (pcDNA-GPR18) resulted in overexpression of the gene that resulted in the production of ROS, NO, and serum lysozyme activity, all crucial for defense against *A. hydrophila* infection [119].

Furthermore, one study produced nine prime-boost vaccines incorporating the recombinant proteins in *Danio rerio* (zebrafish), with varied efficacies of results between the recombinant variations [118]. The vaccines tested consisted of rAHA_2145, rpilQ, rAHA_3766, rgspD, rtamA, rAHA_2785, rAHA_2144, rhgpB, and rAHA_1130. Of these vaccine candidates, the r*AHA_2144* and r*pilQ* resulted in an RPS of 82.4% and 70.2%, respectively, by expressing immune-related genes, resulting in the upregulation of immunological components such as IL-8 and IL-1β, and *Lyz* with the transcriptional expression of *MHCII*, respectively [118]. This upregulation was much stronger following the prime boost; however, it was still present following the initial dose [118]. The other candidates were proven to enhance immunogenicity and RPS to some degree with varying significance [118].

Finally, the incorporation of nanoparticle carriers was present in four different DNA vaccines. This includes the use of single-walled carbon nanotubes in the SWCNTs-aerA vaccine (SWCNTs-pEGFP-aerA), tested in *Ctenopharyngodon idellus* (grass carp); poly-lactic-co-glycolic acid in a vaccine containing the aopB gene (pCDNA3.1-aopB) in *Cyprinus carpio* (common carp); and two chitosan-tripolyphosphate (Cs-TPP) nanoparticle-based vaccines using *OMP* and *hyl* genes (Cs-TPP-pVAX-OMP, Cs-TPP-pVAX-*hly)*, also tested in *Labeo rohita* (Rohu), which will be expanded on further in Section 5.2 regarding nanotechnology, to show the implications of these nanoparticles [117,122,123]. The administration of the SWCNTs-pEGFP-aerA vaccine did, however, show enhancements in immune-promoting genes of TNFα, IL-8, IgM, C-reactive protein (CRP), type I interferon (IFN-I), cluster of differentiation 8 alpha (CD8α), and MHC class I in the kidney cells [122].

Together, these studies represent the new approach of DNA vaccines to introduce cutting-edge gene-specific immunomodulation to elicit specific mechanisms that boost RPS through adaptive and innate immune response to combat *A. hydrophila* infections. This could, however, pose a danger as the fish that receive the vaccines are meant for consumption, leading to possible ethical and health concerns for humans. Despite this, DNA vaccines are generally considered minimally hazardous to the environment and safe for humans and fish [124,125,126,127].

### 3.6. Available Licensed Vaccines for Aeromonas hydrophila

Vaccines against *A. hydrophila* are critical for reducing mortality and enhancing immunity in aquaculture species such as tilapia, carp, and catfish [128]. Licensed vaccines include a range of formulations such as live attenuated, inactivated, subunit, recombinant, and DNA-based vaccines [43]. Examples include AquaVac^®^ Aeromonas by MSD Animal Health, an inactivated or subunit vaccine administered via immersion or injection, and ALPHA JECT^®^ micro 6 by Pharmaq, an injectable inactivated vaccine targeting both *A. hydrophila* and other fish pathogens (Table 1). Other licensed options include bacterins, such as oil-adjuvanted formulations, that provide broad protection against bacterial infections. These vaccines primarily stimulate both humoral and cell-mediated immune responses, leveraging mechanisms like targeted antigen presentation and antibody production to enhance disease resistance [129]. The efficacy of these vaccines is often measured by RPS following pathogen challenges, with various studies indicating high protection rates when optimal administration methods, concentrations, and adjuvants are used [130]. The availability of licensed vaccines offers a reliable tool for mitigating economic losses in aquaculture while promoting sustainable fish farming practices [22].

**Table 1:** Licensed vaccines for *Aeromonas hydrophila*.

Vaccine Name	Manufacturer	Type	Target Species	Administration Route	Licensed Countries	Key Features
AquaVac^®^ Aeromonas	Merck Animal Health	Inactivated	Fish (various species)	Immersion or injection	USA, Canada, Chile, Norway	Effective against multiple *Aeromonas* strains
ALPHA JECT^®^ micro 6	PHARMAQ	Multivalent (inactivated)	Atlantic salmon	Injection	Norway, UK, Chile, Canada	Combines protection against several pathogens
Norvax^®^ Compact PD	MSD Animal Health	Inactivated, multivalent	Salmonids	Injection	Norway, Scotland, Ireland	Targets *A. hydrophila* and other bacterial pathogens
AquaVac^®^ Relera	Merck Animal Health	Inactivated, multivalent	Catfish	Immersion or injection	USA, Brazil, Vietnam	Dual protection against *A. hydrophila* and *E. ictaluri*
*Vibrio*-*Aeromonas S*	Vaxxinova	Inactivated, bivalent	Various fish species	Immersion	Norway, Chile, Japan	Protection against *Vibrio* and *Aeromonas* species

## 4. Immunological Challenges in Vaccine Formulation

The production of a vaccine begins with the decision to produce a strain-specific vaccine for maximum survival rate for aquaculture in a particular region or produce a broad-spectrum vaccine that is much more universal in its application. The formulation of this requires starting with the correct antigenic component that varies significantly with bacteria like *A. hydrophila*, which has extremely high intraspecific genetic variability through the consistent presence of SNPs and unique virulence genes [131,132]. This requires using genomic and proteomic approaches to identify the antigen present in a specific strain, which will be expanded on later. No vaccine developed for *A. hydrophila* to date has proven effective against all strains, as the pathogen’s extensive genetic diversity and adaptability prevent a universal solution. As a result, it is vital to produce vaccines that are tailored to beat these genetic challenges. The use of different adjuvants, delivery systems, and delivery methods may solve some of these problems; however, their broader implications will be expanded on in Section 5. The testing phase for vaccine development relies heavily on environmental stimulation that matches the conditions for a particular aquaculture to produce maximum efficacy results. This includes environmental factors of temperature level, pH level, ammonium concentration, oxygen concentration, diets, and organic matter present in the aquaculture to minimize stress and induce maximum immune response. Following the administration through various delivery methods, testing includes monitoring antibody titers, RPS, and bacterial load post-vaccination in species-specific trials. These results determine the viability of the vaccine and are vital for vaccines tailored for specific farms that struggle with a specific variant strain of *A. hydrophila* that lacks the typical antigenic entities present in a generalized vaccine. These requirements to produce an ideal vaccine are reliant on factors of pathogen diversity, environmental factors, and host factors, which will be expanded on in this section.

### 4.1. Pathogen Diversity

The genomic diversity of *A. hydrophila* is underscored by its open pangenome, comprised of over 18,000 genes, which consists of a core genome shared across all strains and an accessory genome unique to specific isolates [132,133]. The accessory genome contributes to strain-specific adaptations, including enhanced virulence, antibiotic resistance, and environmental survivability. Pangenomic analyses have identified 312 virulence-related genes that impact the effector delivery system, immunological modulation, motility, metabolic factors, adherence, regulation, exotoxin, exoenzyme, invasion, stress survival, biofilm production, antimicrobial activity offering a competitive advantage, and post-translational modification [132]. The variability in virulence gene detection among various isolates could be attributed to the geographical distribution of the strains and the potential occurrence of horizontal gene transfer [133,134,135].

In a study consisting of 31 *A. hydrophila* strains, random amplification of polymorphic DNA (RAPD) and enterobacterial repetitive intergenic consensus (ERIC) molecular techniques were employed to assess genetic diversity, resulting in the identification of 29 unique genotypes [131]. The subspecies of the bacterium were identified through phenotypic diagnostic tests, which involved assessing acid production from salicin, sucrose, and l-arabinose, as well as evaluating the utilization of l-glycine, l-arabinose, and α-methyl d-mannoside as sole carbon sources [131,136]. The genetic diversity within the species was quantified for the first time using RAPD and ERIC scores, with a Nei’s gene diversity index of h = 0.364 ± 0.024 and Shannon’s information index of I = 0.538 ± 0.030, confirming significant intraspecific variability [131]. This genetically heterogeneous species also showed a clear clustering of strains based on their ecological origins [131]. There is also a significant variation in genome sizes, which range from 4.3 to 5.45 Mbp across strains, and in coding sequences, which vary between 4091 and 5134 [132].

*A. hydrophila* isolates carried one or more of the five virulence genes, with *aerA* and *hlyA* coding for enterotoxins, and *alt*, *act*, and *ast* coding for hemolysins, which contribute substantially to fish mortality [15]. The *alt* gene was predominantly found in diarrheic isolates while the *ast* gene was found in almost all isolates of various ecological origins, revealing the selective presence of virulence genes [131]. With this, *A. hydrophila* isolates consistently generate lateral flagella for swarming and surface movement, as well as polar flagella for movement in suspension [1].

The pathogenic diversity of antimicrobial resistance is also a major battleground due to the unregulated use of antimicrobial agents by farmers to control outbreaks within aquaculture [137]. These resistance genes are spread through horizontal gene transfer across pathogenic bacterial species, resulting in at least some level of resistance when presented with 11 different subclasses of antibiotics [133]. A variety of isolates were tested in fish species of tilapia, carp (*Cyprinus carpio*), and channel catfish (*Ictalurus punctatus*), which all reached a multiple antibiotic resistance (MAR) index score averaging 0.747, which is well above 0.2, indicating that these bacterial isolates originated from environments with significant antibiotic exposure, where antimicrobial drugs are frequently utilized, resulting in consistent antibiotic resistance across isolates [133,138,139]. These characteristics of the isolates demonstrate the pathogen’s response to treatment that is consistent through all aquaculture species in showing some degree of resistance.

Along with this, single-nucleotide polymorphisms (SNPs) were identified in the key genes of *speG*, *cheD*, *gmhB*, and *nagL*, which are present in all genotypes of *A. hydrophila*, making them crucial for identification and phylogenetic analyses [132]. The *speG* gene contains seven unique SNPs, while the other genes possess one SNP each, collectively reinforcing the genetic heterogeneity within the species and its genomic adaptability to diverse environments [140]. Studies on the phylogenetic relationships among *A. hydrophila* strains underscore the critical role of SNPs in tracing evolutionary pathways and differentiating closely related genotypes.

These mentioned characteristics of *A. hydrophila*, from its high genetic diversity to adaptability, emphasize the immunological challenges of developing a vaccine. Also, high variability within genetic traits among strains makes it difficult to identify universal targets, while the nature of its highly dynamic genome, driven by HGT and environmental pressures, further complicates efforts at developing a broadly effective vaccine. These are factors that signal the need for innovative approaches that take into consideration the genomic plasticity and adaptability of *A. hydrophila*.

### 4.2. Host and Environmental Factors

Current vaccine studies for *A. hydrophila* typically focus on commonly farmed fish species, which are often specific to the region where the research is conducted. Frequently tested species include grass carp, channel catfish, tilapia, and rohu, as these represent widely cultivated aquaculture species [34]. Most freshwater species exhibit similar susceptibility to the bacterium, with varying bacterial concentrations observed in the muscles, gills, and liver across different species [141]. This is due to host signals of components like blood, serum, and tissue, which can induce the activation of cytotoxic toxins, resulting in the expression of virulence genes [142,143]. However, studying these interactions is challenging due to the complexity of host–pathogen dynamics, the technical demands of transcriptomics and proteomics approaches, and strain diversity [143,144].

Given this, it was found that environmental factors seem to be better understood to predict mortality in *A. hydrophila*’s presence. The bacterial virulence genes of the type III secretion system, elastase, aerolysin, β-hemolysin, hemolysin, flagellin, lipase, cytotoxin, and OmpTS, were present across multiple strains of *A. hydrophila* when tested in *Labeo rohita* [38]. The challenge of specific bacterial isolates revealed an optimal temperature of water at 28 °C for warm-water fish where a majority of these genes were most elevated [38]. This was quantified by the densitometric method, which compared the ratios of each gene product relative to the 16S rDNA product at each temperature tested, where the hemolysin gene, in particular, exhibited a significant level of virulence [38]. These findings reveal the importance of the temperature of the aquaculture to limit the pathogen’s virulence, which can be employed by farmers. The temperature range of the host organism, much like water temperature, can correlate with their susceptibility.

The presence of ammonia can also build up to harmful levels in aquaculture through the metabolism and decomposition of organic matter from aquatic organisms [143]. These high ammonia levels exacerbate *A. hydrophila* infections by weakening host immunity and fostering conditions that promote bacterial growth. One study found that an ammonia concentration of 0.3 mg/L in Nile tilapia reduced mortality when tested independently [145]. The pH level of the water was also vital, with an optimum level between 6.5 to 8.5 for tilapia, where the ammonia present changes into the form of ammonium ions, which are significantly less toxic to the aquaculture at hand [143,146]. pH levels outside of this range deionize the ammonia, significantly increasing mortality [143,147,148]. Much like temperature, irregularities with ammonia conditions also lead to the upregulation of specific *A. hydrophila* virulence genes, such as the *fla* gene in the tilapia study [145].

Another key environmental factor is oxygen concentration, impacting virulence by regulating gene expression through transcription factors like FNR and NarL [149,150]. Under low oxygen levels, FNR activates virulence and anaerobic respiration genes, while oxygen deactivates it by altering its iron–sulfur cluster [149]. Additionally, oxygen indirectly affects virulence by influencing reactive oxygen species (ROS) and NO levels, which bacteria sense and counteract through enzymes such as superoxide dismutase, catalases, and NO reductases to modulate virulence gene expression [143,150,151]. The last environmental component is the presence of organic matter as excess feed and eutrophication can lead to rapid bacterial multiplication, increasing disease outbreaks, while specific supplements like NSS microalgae blend and omega-3-rich diets have shown promise in boosting fish immune responses and reducing mortality rates, though more research is needed to clarify these relationships [1,145,152,153]. The specifics between fish species may change; however, it is known that the accumulation of these suboptimal conditions promotes stress in aquaculture that has been shown to weaken innate and adaptive immune responses. This suppression of the immune system makes these aquaculture species much more susceptible to the proliferation of the bacterium.

Testing of a variety of antibiotic classes found that tilapia isolates exhibited higher resistance to isolates from carp and channel catfish, which were generally more susceptible to antibiotics like neomycin, cephalosporins, fluoroquinolones, and sulfonamides, suggesting that these host species may experience lower selective pressures for resistance [133]. These variations in resistance patterns among fish species may reflect differences in the frequency, quantity, and methods of antimicrobial use across fish culture systems, as well as geographic differences in resistance [133,154,155,156]. The MAR index values also varied across species, with 87.6% of the isolates from tilapia, 73.3% in carp isolates, and 63.3% in channel catfish isolates [133]. Further testing through other freshwater species would indicate further variations in the results of antibiotic resistance because of the host organism at hand.

## 5. Innovations in Vaccine Development

Recent advancements have led to innovative approaches to enhance vaccine efficacy. This includes the use of genome sequencing to identify the most conserved and virulent genes for DNA vaccines, as well as proteomics to profile possible antigenic entities to be used as immunogenic targets in recombinant or subunit vaccines, given factors of strain variability. These approaches are coupled with the incorporation of nanotechnology, as once the vaccine is produced, they offer protection through encapsulation, increasing targeting and the release of the immunogenic components. The selection of one or multiple adjuvants is also vital as it acts as an immunostimulant for the incorporation of DNA or antigens. The final variable approach in vaccine development is the delivery method, such as the less invasive method of bath immersion and oral delivery, as well as intramuscular and intraperitoneal injections, which show various efficacies in different situations. The details surrounding these factors and challenges will be expanded in this section.

### 5.1. Genomic and Proteomic Approaches

Whole-genome sequencing of *A. hydrophila* has been instrumental in uncovering genetic components involved in pathogenicity and immune evasion. This approach enables the identification of novel virulence, including genes encoding cytotoxic enterotoxins (Act), hemolysins such as aerolysin and *hlyA*, exotoxins homologous to Exotoxin A, adhesion molecules like lateral and polar flagella, and components of secretion systems (T3SS, T2SS, T6SS) that facilitate toxin delivery, adhesion, and immune evasion [157,158,159,160,161]. For vaccine development, whole-genome sequencing allows researchers to pinpoint highly conserved genes across multiple *A. hydrophila* strains. The genomic analyses of *A. hydrophila* and its phages integrate high-throughput sequencing platforms like Illumina, PacBio, MinION nanopore technology, Roche 454, and the GS Junior pyrosequencer for genome sequencing; genome assembly through tools like SPAdes, BWA, and Newbler; gene prediction with Glimmer 3.0 and Prodigal; annotation using Prokka, RAST, and BLASTP/RPS-BLAST for the NCBI Conserved Domain Database (CDD) and COG; variant analysis using GATK; functional trait exploration through tRNAscan-SE and Artemis; and long-range scaffolding and enhanced assembly accuracy through MinION reads, allowing for the characterization of virulence factors, prophages, antibiotic resistance, and functional genes [157,158,159,160,161]. The identification of strain-specific antigens facilitates the development of customized vaccines for specific aquaculture environments or host species. Phylogenetic analyses in these studies utilize tools like MEGA for constructing neighbor-joining trees, core genome-based phylogeny, and ANI-based clustering, supported by genome-to-genome distance calculations (dDDH), MUSCLE for sequence alignment, and visualization platforms like iTOL, enabling the classification of *A. hydrophila* strains, identification of taxa, and insights into evolutionary relationships [157,158,159,160,161].

Proteomic approaches complement genomic studies by decoding molecular responses of *A. hydrophila* during infection and stress. Techniques like iTRAQ, SWATH-MS, and MALDI-TOF identify differentially expressed proteins linked to immune evasion, metabolic adaptation, and antibiotic resistance [162,163,164,165]. These proteomic methods allow for the detailed characterization of stress-related and immunogenic proteins, offering critical insights into bacterial survival mechanisms, such as upregulated translation processes under antibiotic stress and downregulated energy pathways during metabolic adaptations [162,163]. Proteomic data are further processed using in silico tools such as BLASTP and RPS-BLAST, which annotates proteins against databases like NCBI, CDD, and COG to uncover virulence factors and metabolic pathways [162,163,164,165]. Visualization and statistical tools, including Artemis, IGV, STRING, and DAVID, are employed to map gene ontology categories, analyze protein–protein interactions, and validate enriched pathways, such as oxidative stress response and immunomodulation during infection [162,163,164,165]. There are several free *A. hydrophila* antigen databases, which include NCBI, BLAST, UniProt, IEDB, PATRIC, PubMLST, and AlphaFold Protein Structure Database, for consolidating, structuring, molecular typing, and prediction.

By integrating genomic and proteomic data, researchers can create comprehensive antigen databases for *A. hydrophila*. These databases enable the rational design of DNA vaccines, subunit vaccines, and attenuated live vaccines that specifically target highly immunogenic and essential bacterial components. For example, genomic analysis has facilitated the identification of plasmid-encoded virulence genes, which have been incorporated into DNA vaccine constructs to stimulate adaptive immunity. Meanwhile, proteomic studies have guided the selection of proteins for recombinant vaccines, offering safer alternatives to whole-cell vaccines.

### 5.2. Nanotechnology

Carrier systems primarily function to deliver the immunostimulant effectively to immune cells through micropinocytosis, phagocytosis, and endocytosis without necessarily providing direct immune stimulation, unlike traditional adjuvants that actively enhance immune activation [166]. These carriers require nanoparticles to encapsulate and are often used for recombinant protein or DNA vaccines, specifically because whole cells in vaccines are about 0.5–5 μm in diameter, making them too large for most nanoparticles [113]. Variations of carrier systems include nanoparticles such as single-walled carbon nanotubes (SWCNTs), Polylactic-Co-glycolic Acid (PLGA) nanoparticles, chitosan-tripolyphosphate (Cs-TPP) nanoparticles, calcium phosphate nanoparticles, immune-stimulating complexes, and others such as multi-walled carbon nanotubes (MWCNTs), which improve vaccine uptake by protecting antigens from degradation through encapsulation or promoting biocompatibility and mucoadhesive properties [34,37,110,113,123,167,168]. Nanoparticles can be designed to mimic pathogen characteristics, including size and charge, enhance retention and biodistribution in lymphoid organs, and be readily internalized by APCs for antigen presentation via MHC-I and II molecules [37].

There continue to be advancements in nanotechnology variations such as liposome nanotubes, dendrimers, and nanocapsules; however, these currently used examples in *A. hydrophila* vaccines will be explored [37]. The use of single-walled carbon nanotubes is at the forefront of vaccination and has been tested in a DNA plasmid vaccine (SWCNTs-pEGFP-aerA) administered through intramuscular injection in *Ctenopharyngodon idella* (grass carp) to test the nanotechnology’s efficacy [122]. This was compared with the same vaccine that lacked the SWCNTs, with the results of a significantly higher survival rate of 83.7% when compared to 45.1% for the equivalent dosage of the vaccine without the use of nanotubes due to a higher yield of plasmid DNA released, resulting in more antigens expressed [122]. The immunological mechanisms and biochemical implications for this study are detailed in Section 3.5 on DNA vaccines. A similar recombinant vaccine study tested four iron-related proteins, P55870, A0KGK5, A0KPP0, and A0KIY3 [169]. The findings showed an increase in RPS when these proteins underwent SWCNT-encapsulation with bath immersion immunization when compared to their nonencapsulated and intraperitoneally injected counterparts [169]. Ammonium-modified MWCNTs were also shown to boost the delivery and expression effectiveness of plasmid DNA in kidney cells of *Ctenopharyngodon idellus* (CIK) in vitro [122,170].

PLGA-based nanoparticle delivery systems were used in three *A. hydrophila* DNA and recombinant vaccine studies as biodegradable, nontoxic encapsulation vehicles. The first two studies consisted of PGLA nanoparticles with a diameter of 370–375 nm, and the final mentioned study had particles averaging 423 nm in diameter [113,117,171]. To start, an orally delivered recombinant OMPW vaccine used PLGA nanoparticles with an encapsulation efficiency of 53% and a zeta potential of 19.3 mV, according to a particle analyzer [113]. The results of this study showed clear protective immunity, with a high RPS of 79.99% for the high-antigen group, despite lower encapsulation efficiency than expected [113]. Another study used PGLAs for a recombinant maltoporin vaccine that showed an encapsulation efficiency of 63%, with a release of 55% of the 60.39 kDa protein within 48 h [171]. This study did not test the vaccine in fish; however, it showed that the PLGA-maltoporin particles had a consistent spherical shape and were evenly dispersed, possibly inducing the best results for immune response [171]. Finally, the last study compared the previously mentioned pCDNA3.1-aopB DNA vaccine with and without encapsulation [117]. The results of the PGLA group displayed increases in innate immune responses of serum lysosome activity, the number of phagocytic cells and their functionality, globulin levels, serum protein levels, and Nitroblue Tetrazolium reduction [117]. Along with this, an elevation in serum antibody titer was observed [117]. This was accompanied by a mortality rate of 20% for the nano-encapsulated group and 35% for the group without encapsulation [117]. These studies together reveal the enhanced potential of vaccines using PGLA encapsulation.

There has only been one study that tested Cs-TPP nanoparticles in two DNA vaccines, Cs-TPP-pVAX-OMP and Cs-TPP-pVAX-*hly*, in *Labeo rohita* (Rohu), as previously mentioned [123]. The oral delivery of the vaccine through fish feed revealed that these nanoparticles promoted humoral and cell-mediated immunity without adjuvants [123,172]. These particles were 200–400 nm with about an 80% encapsulation efficiency, where clear stained DNA release was present when chitosanase digestion was introduced, promoting expression of the OMP and *hyl* genes [123,173,174]. The nanoparticles facilitated B cell conversion to IgA synthesis through upregulated TGFβ expression, resulting in improved antibody production and microbial clearance. They also promoted increased lysozyme gene expression, significantly improving RPS to 76.2% in challenged Rohu [123,175,176]. An S-layer protein-based vaccine study in *Labeo rohita* (Rohu) used calcium phosphate nanoparticles due to their low cost and proven efficacy [177]. The nanoparticles directly maintained high antibody responses up to 63 days post-immunization and significantly increased respiratory burst, neutrophil, and bactericidal activities compared to other formulations [177]. When the Rohu were challenged with different strains of *A. hydrophila*, the vaccine provided 100% protection without altering serum calcium or phosphate levels through bioaccumulation [177]. The final type of nanoparticle is the immune-stimulating complex (ISCOM), used in a major outer membrane protein (MOMP) recombinant vaccine [178]. This component does not serve the same encapsulation function as the previously mentioned nanotechnologies; however, it is worth mentioning as it resulted in an 80% RPS and was shown to be a key protective antigen, resulting in immunogenic protection in challenged fish [178].

These carriers also face challenges such as a high initial release, incomplete antigen delivery, protein instability, and potential bioaccumulation risks in the environment with nonbiodegradable particles leading to nanotoxicity within gills, liver, and brain tissues, leading to oxidative stress [37,113]. This stress could increase the susceptibility of other pathogenic bacteria and could be induced by the other nanoparticles mentioned as well. Despite this, nanoparticles overall reveal improvements in vaccine efficacy and should be further studied to apply in other struggling vaccines due to their properties in stimulating targeted immunity.

### 5.3. Advanced Adjuvants and Delivery Systems

Adjuvants in *A. hydrophila* vaccines are vital to the success of a vaccine as well. They are not necessary for live attenuated *A. hydrophila* vaccines as these microbial vectors often already contain properties of adjuvants, with their ability to replicate and resemble a natural infection [179]. They can act as immunostimulants that work in conjunction with immunogenic agents present in vaccines, such as variations of OMPs, lipopolysaccharides, extracellular proteins, *aerA* proteins, and S-layer proteins to enhance immune response [34,37,180,181,182]. Mechanistically, some adjuvants involve the adaptive immune system by activating APCs through MHC molecules, which then trigger B and T cell responses [37]. This helps to boost immune memory and pathogen resistance in fish [37]. *A. hydrophila* vaccines specifically utilize more conventional adjuvants and carrier systems, while nanotechnology carriers and herbal-based adjuvants have been applied and tested significantly less [34]. The common adjuvants that are used across almost all current *A. hydrophila* vaccines include Montanide ISA (non-mineral oil), Montanide adjuvant, aluminum hydroxide, Freund’s adjuvant, PBS-mineral oil, ISA 763, plasmid-encoded cytokine adjuvant, and QCDCR adjuvant [34,79,92,93,94,95,96,97]. These compounds exist in a variety of vaccines and are predominantly used in subunit, recombinant, and DNA vaccines [34].

Along with this, the herbal adjuvant of *Asparagus racemosus* extracts has been tested in a subunit vaccine developed in India, as previously noted in *Carassius auratus* (goldfish), leading to an RPS of about 50% [93]. The *A. racemosus* adjuvant showed strong immune-stimulating effects, especially at the humoral level in experimental settings. Its main active component, saponins, encourages peripheral lymphocyte proliferation, increases serum antibody levels, and provides a safer alternative to chemical adjuvants [93]. Further holistic approaches like this will be detailed in Section 6.2. Although there have been consistent results of efficiency from these common adjuvants, it has been discovered that adverse effects of their incorporation include adhesions, granulomas, and chronic peritonitis in some extreme cases [103,110,183,184].

The delivery system of these vaccines is also vital to their efficacy as they are administered to aquaculture through oral delivery, intramuscular and intraperitoneal injections, and immersion [34]. Intraperitoneal injections were the most common delivery method used in *A. hydrophila* studies, while intramuscular injection was often used in DNA vaccines [34]. These injection delivery methods are challenging as they induce stress, require substantial labor, and have higher costs, which makes them suitable primarily for larger fish [34,105,185]. This can also be problematic for mortality rates as *A. hydrophila* infections pose a threat to fish that are immunocompromised or under stress. Despite this, the large amount of data across these studies shows that intraperitoneal injection offers the highest protection in the fish tested due to benefits such as requiring lower antigen doses, achieving high vaccination efficacy, and boosting immunogenicity through the use of adjuvants [84,186]. Injection methods are unsuitable for lower-value and small-sized fish species, making bath immersion and oral administration the often-preferred route for aquaculture farmers.

Immersion delivery systems, through mucosal routes, simplify antigen administration and cause less stress compared to injection-based methods [84]. In this method, fish are immersed in a vaccine solution for a set duration, allowing antigens to be absorbed through mucosal surfaces [88,187]. Dose concentration, length of protection, timing of delivery, initial vaccination size, booster schedules, and storage conditions are vital to the antigen uptake in hatcheries [187]. Despite this, bath immersion has shown poor antigen uptake through the gills and skin for the recombinant *A. hydrophila* vaccine specifically [34,110]. SWCNTs-aerA DNA and recombinant vaccines resulted in high RPS through bath immersion, oral delivery, and intramuscular injection due to the substantial loading capacity of functionalized SWCNTs, which provides numerous binding sites for additional chemical modifications with biologically active compounds [105,188]. The high antigen-loading capacity of CNTs makes them suitable as delivery vectors for recombinant and subunit vaccines, potentially enabling effective bath immunization (immersion) in smaller fish and offering a more sustainable solution for delivery methods [105,189].

Oral delivery was shown to be the most effective in live attenuated and nanoparticle-encapsulated vaccines due to the protection of the antigen, whereas recombinant, subunit, DNA, and inactivated vaccines have been shown to have issues with antigen incorporation as degradation occurs from the acidity of the foregut [34,190]. They are often presented in feed, reducing stress, bioaccumulation, and labor. Overall, antigen uptake is generally inefficient with oral delivery systems, as most *A. hydrophila* vaccines consist of unencapsulated antigens. Despite this, there have been plenty of studies that have shown significant vaccine efficacy with this delivery system regardless of the vaccine type.

## 6. Future Perspectives

The future of managing *A. hydrophila*-related challenges in aquaculture lies in integrated, targeted, and sustainable solutions. Personalized vaccines tailored to specific farm environments and strains offer a practical and effective pathway for reducing disease outbreaks. By employing genomic and proteomic tools, researchers can identify highly immunogenic, conserved antigens, enabling the design of vaccines that maximize immune response while accounting for the pathogen’s genetic diversity. Innovations such as nanoparticle-based delivery systems and plant-based adjuvants are anticipated to enhance vaccine stability, safety, and accessibility, reducing reliance on traditional methods with higher environmental costs. Moreover, holistic approaches like Integrated Disease Management (IDM), which combine biosecurity, optimal water quality, dietary interventions, and alternative therapies, including probiotics and herbal extracts, promise to reduce antibiotic dependency and promote fish health.

Scaling these solutions will require a concerted effort to lower production costs and improve distribution in low-resource settings. Modular production units, localized vaccine manufacturing, and feed-based vaccine formulations can mitigate economic barriers, ensuring accessibility for small-scale farmers. Furthermore, advancements in eco-friendly vaccine components, such as self-destructing recombinant attenuated vaccines and biodegradable delivery systems, can minimize ecological impacts, aligning with sustainable aquaculture practices. Collaborative industry and scientific partnerships will be crucial in addressing existing bottlenecks and driving innovation. Ultimately, integrating vaccination with sustainable aquaculture practices will foster resilience against *A. hydrophila*, ensuring food security, economic viability, and environmental stewardship in the global aquaculture sector.

### 6.1. Personalized Vaccines

*A. hydrophila* generally produces highly conserved antigenic components among different strains despite having heterogeneous biochemical and serological features, providing cross-protection against various isolates [77,191,192]. However, finding epidemiological units of pathogenicity to produce broad-spectrum vaccines generally makes their use only financially feasible for fish species of high economic value [34]. Given this, farm-specific and autogenous vaccines, where *A. hydrophila* is rampant, are the most economical for low- and middle-income countries that produce much of the world’s aquaculture [34]. The developmental efforts require a more narrowed approach as a conserved antigen does not need to be identified. This combats the challenges of strain variability where alterations of antigenic sites frequently occur, providing targeted protection.

The autogenous vaccine starts by directly removing the pathogen from the aquaculture at hand and applying genomics and proteomic analyses to identify a virulent antigen or produce whole-cell variants present. Following the formulation, the vaccine is delivered through various delivery methods, with different adjuvants tested in the specific species of aquaculture. The RPS following the challenge would indicate the best formulation and delivery method, which should demonstrate consistency and effectiveness under field conditions. A consequence of a viable vaccine that is tailored to a specific strain, host factors, and geographical region is a decrease in the use of medications such as antibiotics, limiting the antimicrobial-resistant properties of the strain.

### 6.2. Integrated Disease Management

IDM strategies include vaccination, biosecurity, improved nutrition, and water quality management, while leveraging herbal treatments, probiotics, and advanced biocontrol techniques. Holistic approaches have been extensively researched and emphasize the integration of vaccination with other disease control measures. Improved water quality and proper nutrition, paired with herbal treatments, have been shown to reduce the pathogenicity of *A. hydrophila* while enhancing fish immunity. Our lab developed a novel approach to construct a recombinant attenuated *Edwardsiella* vaccine (RAEV) with a unique biological containment phenotype that induces regulated bacterial cell wall lysis. This mechanism ensures that the vaccine strain cannot persist in the environment. The RAEV is the first live attenuated vaccine candidate designed specifically for the aquaculture industry to combine the containment property with sensitivity to all antibiotics, making the vaccine both environmentally responsible and treatment-compatible [88]. Medicinal plants containing bioactive compounds, such as flavonoids, terpenoids, and alkaloids, exhibit antimicrobial, immunostimulatory, and anti-stress properties [1]. Studies have highlighted phytotherapy as a sustainable alternative to synthetic pharmaceuticals for disease management in aquaculture [1]. Similarly, the efficacy of combining herbal extracts like *Boesenbergia pandurata*, *Solanum ferox*, and *Zingiber zerumbet* in boosting survival rates and reducing bacterial loads in tilapia has been demonstrated [193]. Macroalgae also showed potential as a natural treatment option in IDM strategies. *Sargassum vulgare* ethanolic extract was identified as an effective antibacterial agent as incorporating macroalgal extracts into fish diets enhanced growth performance and reduced bacterial loads [194].

Probiotic- and prebiotic-enriched diets are fundamental components as diets supplemented with *Lactobacillus* and triherbal extracts significantly improved immune responses, reducing mortality in carp. Probiotic treatments enhanced respiratory burst activity and serum-mediated bacterial killing while maintaining stable glucose and protein levels [195]. Additionally, functional feeds enriched with probiotics, essential oils, and algae-derived compounds were found to enhance fish immunocompetence and decrease antibiotic dependency, which is vital, given the resistant nature of the bacterium [196]. Bacteriophage therapies, a form of biocontrol targeting multidrug-resistant *A. hydrophila*, have demonstrated promising results by utilizing isolated bacteriophages, such as ΦZH1 and ΦZH2, to effectively reduce bacterial populations and control MAS [197]. Combining natural flavonoids like rutin with antibiotics such as florfenicol has been shown to reduce oxidative stress and enhance immune responses in tilapia as well [198]. Several other studies are testing the efficacies of phages, herbal extracts, etc., with varying implications on immunogenic capabilities; however, the consensus promotes these methods as a facet of IDM.

Bivalent and multivalent vaccines play a critical role in aquaculture disease management by targeting *A. hydrophila* alongside other pathogens, including *Vibrio vulnificus*, *Vibrio fluvialis*, *Vibrio anguillarum*, *Edwardsiella anguillarum*, *Streptococcus agalactiae*, *Streptococcus iniae*, *Aeromonas veronii*, and *Lactococcus garvieae* [35,108,199,200,201,202,203,204]. These vaccines leverage a range of technologies, such as recombinant vaccines, live attenuated vaccines, DNA vaccines, secretory antigen delivery systems, and feed-based or formalin-inactivated cell preparations, achieving viable RPS rates through most studies [35,202,203,204]. Despite these successes, challenges persist, including the difficulty of identifying conserved antigens across highly variable bacterial strains, which limits their broad efficacy [202,203,204]. Antigenic competition within formulations can suppress immune responses to certain components, undermining protection against co-targeted pathogens [35,203]. These issues, coupled with higher production costs and the complexity of scaling up, hinder their practical use [35,204]. Studies that overcome these challenges position multivalent vaccines as key tools in reducing antibiotic reliance while maintaining a sustainably healthy population.

The real key, aside from additives, is that farming practices like managing stocking densities, monitoring diseased fish, and maintaining water quality are critical in preventing stress-induced disease outbreaks. The importance of biosecurity and environmental controls in managing bacterial infections in ornamental fish, particularly in high-density farming environments, is evident [205]. Incorporating these approaches alongside advancements in genetic modification and selective breeding offers a comprehensive IDM framework. This integration improves fish resistance, reduces disease spread, and promotes sustainable aquaculture practices [194,196].

### 6.3. Sustainable and Cost-Effective Solutions

Innovations in the manufacture of vaccines have targeted cost reduction to ensure affordability for small-scale and resource-poor farmers. Techniques like local production in low-resource settings contribute to eliminating reliance on costly imports and addressing barriers to vaccine accessibility [206]. Process optimization, infrastructure utilization, and modular production facilities will be important strategies for efficient scale-up while minimizing costs [207]. Recombinant vaccines have emerged as a cost-effective solution, utilizing microbial fermentation systems to produce antigens efficiently and affordably [207]. Alongside this, feed-based vaccine formulations further reduce costs by eliminating the need for labor-intensive delivery methods such as injections, streamlining both production and administration processes [208].

Sustainable vaccine development considers minimal environmental impact in conjunction with aquaculture practice that reduces the use of antibiotics. Advances in the stabilization of vaccine formulations have reduced cold-chain dependency, which decreases energy-intensive logistics and increases shelf-life stability [206]. Furthermore, environmentally friendly adjuvants and biodegradable delivery systems, are in development to reduce waste and ecological contamination [207]. However, challenges persist in assuring these sustainable practices at economically viable scales, especially among small-scale aquaculture operations.

## 7. Conclusions

*A. hydrophila* remains a significant threat to global aquaculture, causing high mortality rates, economic losses, and contributing to the growing challenge of antibiotic resistance. Vaccination has emerged as a sustainable alternative to antibiotics, offering a promising solution to manage infections while safeguarding fish health. Advances in vaccine technologies, including live attenuated, inactivated, subunit, recombinant, and DNA-based approaches, have shown considerable potential in stimulating robust immune responses. Innovations such as genomic and proteomic tools, nanoparticle delivery systems, and plant-based adjuvants are paving the way for more targeted and effective vaccines tailored to diverse aquaculture environments. Cutting-edge technologies like the RAEV, which combines environmental containment with sensitivity to all antibiotics, highlight the dual benefits of safety and compatibility with standard treatment protocols. This innovation exemplifies the strides being made toward more sustainable and effective vaccine solutions for the aquaculture industry.

Despite these advancements, challenges persist, including strain variability, high production costs, and the complexity of vaccine delivery. IDM strategies that combine vaccination with improved biosecurity, water quality management, and alternative therapies offer a holistic approach to disease prevention. Scaling vaccine accessibility and affordability through localized production and innovative delivery methods will be critical to meeting the needs of small-scale aquaculture operations. Moving forward, industry-wide collaborations, enhanced funding, and sustainable practices will be essential to translate research breakthroughs into scalable, practical solutions. By addressing these challenges, *A. hydrophila* vaccination programs can significantly contribute to sustainable aquaculture practices, ensuring food security and economic resilience globally.
